# Spatial chloroplast-to-nucleus signalling involving plastid–nuclear complexes and stromules

**DOI:** 10.1098/rstb.2019.0405

**Published:** 2020-05-04

**Authors:** Philip M. Mullineaux, Marino Exposito-Rodriguez, Pierre Philippe Laissue, Nicholas Smirnoff, Eunsook Park

**Affiliations:** 1 School of Life Sciences, University of Essex, Wivenhoe Park, Colchester CO4 3SQ, UK; 2 College of Life and Environmental Sciences, Biosciences, University of Exeter, Exeter EX4 4QD, UK; 3 Plant Immunity Research Center, College of Agriculture and Life Sciences, Seoul National University, Seoul 08826, Republic of Korea; 4 Department of Molecular Biology, College of Agriculture and Natural Resources, University of Wyoming, Laramie WY 82071, USA

**Keywords:** retrograde signalling, hydrogen peroxide, plastid–nuclear complexes, stromules, gene expression

## Abstract

Communication between chloroplasts and the nucleus in response to various environmental cues may be mediated by various small molecules. Signalling specificity could be enhanced if the physical contact between these organelles facilitates direct transfer and prevents interference from other subcellular sources of the same molecules. Plant cells have plastid–nuclear complexes, which provide close physical contact between these organelles. Plastid-nuclear complexes have been proposed to facilitate transfer of photosynthesis-derived H_2_O_2_ to the nucleus in high light. Stromules (stroma filled tubular plastid extensions) may provide an additional conduit for transfer of a wider range of signalling molecules, including proteins. However, plastid–nuclear complexes and stromules have been hitherto treated as distinct phenomena. We suggest that plastid–nuclear complexes and stromules work in a coordinated manner so that, according to environmental conditions or developmental state, the two modes of connection contribute to varying extents. We hypothesize that this association is dynamic and that there may be a link between plastid–nuclear complexes and the development of stromules. Furthermore, the changes in contact could alter signalling specificity by allowing an extended or different range of signalling molecules to be delivered to the nucleus.

This article is part of the theme issue ‘Retrograde signalling from endosymbiotic organelles’.

## 1. Introduction

In eukaryotes, the nucleus is the recipient of intracellular signals from every other organelle and compartment [1], which strongly suggests that spatial (i.e. three dimensional) as well as temporal components in signalling networks are of the utmost importance in terms of signalling specificity and the determination of cell fate. The continual adjustment to cellular metabolism in a fluctuating environment, which every photosynthetically active plant cell in a leaf has to carry out, depends upon the communication from its chloroplasts to the nucleus (hereafter termed retrograde signalling). Conversely, adjustments to primary metabolism involve much communication from the nucleus to plastids (termed anterograde signalling [1]) and can result in changes to photosynthesis, alter protective mechanisms such as antioxidant capacity and modulate hormone biosynthesis.

In this short paper, we have not considered mitochondrion–nucleus retrograde signalling. Instead, we refer the reader to other articles in this special issue. Rather, we have focussed on two means by which physical contact between plastids and the nucleus have been reported: plastid–nuclear complexes and stromules. We consider what is known about the dynamics of these interactions, the implications of close proximity of these organelles for the specificity of retrograde signalling as raised previously [2–4], and begin to consider the notion that sub-populations of chloroplasts may have distinct cellular functions.

## 2. Plastid-nuclear complexes in higher plants and algae

**Figure 1.**
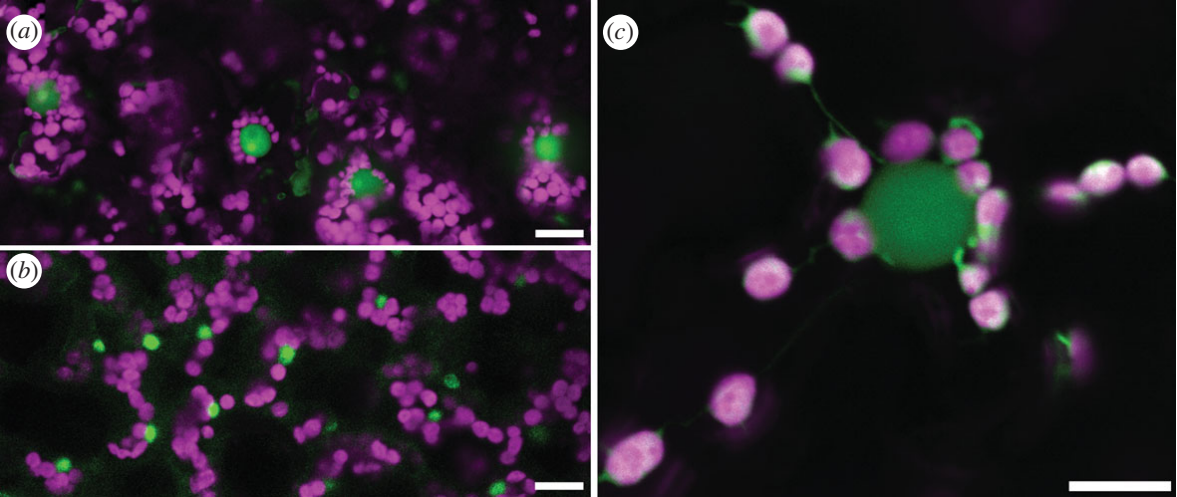
Nucleo-plastid association in *Arabidopsis thaliana* and *Nicotiana benthamiana*. All chloroplasts are magenta, all nuclei green. All scale bars 10 µm. (*a*) Nuclei are surrounded by chloroplasts in the typical 'daisy flower' arrangements in *N. benthamiana* abaxial epidermal cells. (*b*) In the spongy mesophyll of *Arabidopsis*, nuclei are in contact with but not surrounded by chloroplasts. (*c*) A nucleus with surrounding chloroplasts from *N. benthamiana* abaxial epidermal cells, displaying occasionally observed stromules under low light conditions.

A close association of plastids, including chloroplasts, and nuclei has been observed in many higher plant species ranging from horsetails (*Equisetum* sp.) to eudicots and monocots ([2]; figure 1*a*,*b*). Plastid-nuclear complexes may have a complex but ordered structure because in some images, the peri-nuclear endoplasmic reticulum may be seen to interpose between chloroplasts and their nucleus [2]. Furthermore, an extensive survey of the positioning of plastids around the nuclei of tobacco epidermal cells strongly suggests a specific positioning between the organelles—the most striking and common being a daisy flower arrangement of plastids associated with the ‘equator’ of the nucleus [2]. This is an arrangement we have also readily observed (figure 1*a*; electronic supplementary material, Movie S1). This apparently precise arrangement could mean that the structure of plastid–nuclear complexes is under tight regulation and amenable to genetic analysis (see below). Algal cells have from one (e.g. *Chlamydomonas*, *Ostreococcus*) to many chloroplasts. In *Chlamydomonas*, the nucleus is enveloped within the cup-shaped chloroplast. In *Ostreococcus tauri*, TEM electron cryotomography shows close association of chloroplast and nucleus with the peroxisome sandwiched between them. At some points during cell division, elongated nuclear envelope processes stream around the chloroplasts [5]. Since *Ostreococcus* cells are very small, with one copy of each organelle, it is difficult to determine if there are specific physical links. Photosynthetic protists of various kinds have chloroplasts derived from secondary endosymbiosis with algae and therefore have more complex membrane arrangement with three to four membranes enclosing the chloroplast and sometimes enclosing a nucleomorph (remnant of the symbiont's nucleus) [6]. Attachment of the chloroplasts to each other and to the nucleus has been reported in *Euglena*, particularly during cell division [7]. Chromosomes are prominent near the contact points. In *Ochromonas*, the nuclear envelope appears to be continuous with the outer chloroplast membrane, with little or no cytoplasm between them [8]. Clearly, more extensive data are needed to assess the extent of chloroplast–nuclear attachments in algae and photosynthetic protists.

The study by Selga *et al*. [2] described plastid–nuclear complexes in 10 plant species that included horsetail, a fern, gymnosperms, eudicots and monocots. This survey suggests that plastid–nuclear complexes in plant cells are the norm, but questions arise about the dynamic nature of plastid–nuclear complexes. For example, is there a turnover of chloroplasts associated with the nucleus? Despite a range of microscopic methods having been applied to image plastid–nuclear complexes [2], we have no impression of their turnover. In many photosynthetic cell types packed with chloroplasts (e.g. estimates of 50–70 in *Arabidopsis* mesophyll cells; [9]) only a proportion of chloroplasts would be able to engage in direct interactions with chloroplasts (figure 1*b*) but it would be difficult to observe turnover. However, in cell types with lower numbers of chloroplasts such as the abaxial epidermal tissue of *Nicotiana benthamiana*, a single time point sampling revealed 3–12 chloroplasts in contact with the nucleus (figure 1*a*,*c*; [4]). This could imply a stochastic process but equally could be reflecting turnover in chloroplast numbers in different plastid–nuclear complexes such that at any timepoint different cells display differing numbers of chloroplasts interacting with their nucleus. Resolving this would require long-term observations of the same cell with differentially marked chloroplasts.

## 3. Plastid-nuclear complexes and the actin cytoskeleton

In the streptophytes, chloroplasts and nuclei move to anticlinal sides of cells away from high fluence blue light such as in the high light (HL) conditions used by the authors [4]. This is called the avoidance response and is controlled by phototropins [10]. The avoidance response of chloroplasts depends upon their interaction with the actin cytoskeleton [3,10,11]. Chloroplasts and the nucleus in each cell are tethered to one another via the actin cytoskeleton and the action of at least three proteins CHLOROPLAST UNUSUAL POSITIONING1 (CHUP1), KINESIN-LIKE PROTEIN FOR ACTIN-BASED CHLOROPLAST MOVEMENT1 (KAC1) and KAC2, which are associated with the plastid outer membrane [3,12–14]. These proteins serve primarily to anchor chloroplasts to the plasma membrane but appear also to be crucial for correct tethering of nuclei to chloroplasts. Nuclei have no independent capacity to move along the actin cytoskeleton, instead relying on their physical association with chloroplasts [3,14]. However, in mutants defective in one or more of these proteins, nuclei still do move in response to incident light, albeit in an unusual manner. This is because in *chup1* and *kac1kac2* mutants, nuclei retain some connectivity to chloroplasts and therefore some capacity to carry out avoidance. Even a triple mutant (*chup1kac1kac2*), while showing severe attenuation, did display some highly aberrant nuclear avoidance responses, implying there was still some nuclear–plastid connectivity [3,14]. Interestingly and in contrast to these mutants, some plastid division mutants (*plastid division1* (*pdv1*)/*pdv2* double mutant and *paralog of arc6* (*parc6*)) are also completely defective in tethering of chloroplasts to the nucleus [3]. In the case of *parc6*, the phenotype shows cell-autonomous behaviour with respect to this phenotype [3] meaning that most cells display a lack of chloroplast-to-nucleus tethering, but a proportion of them do not. Therefore, it may prove possible to compare cells with nuclei attached to chloroplasts alongside cells with separated chloroplasts and nuclei in the same tissue. This may obviate issues around the possibility of pleiotropic effects of such mutants. While there are many questions surrounding the use of *chup1*, *parc6* and other such mutants, they do indicate both the complexity and the likely dynamic nature of these plastid–nuclear complexes. In summary, we conclude that plastid–nuclear complexes are unlikely to be static structures and in considering their interactions with the cytoskeleton and overlap with plastid division they share commonality with stromules (see below).

**Figure 2.**
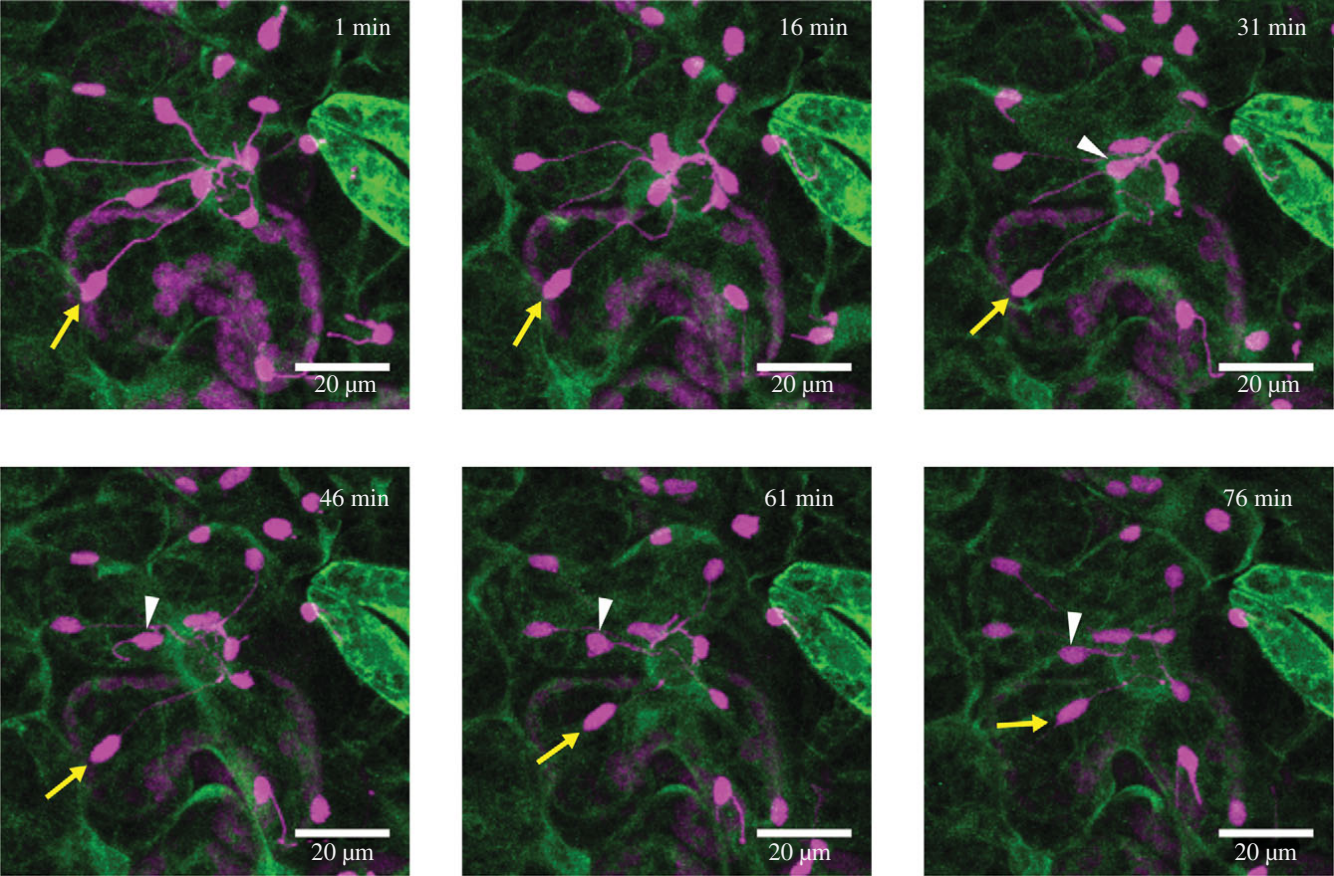
Stromule-mediated perinuclear clustering (PNC) of the chloroplast during plant immune responses. NUCLEAR RECEPTOR INTERACTING PROTEIN 1 (NRIP1)(cTP)-TagRFP (magenta) were transiently expressed to visualize chloroplasts and stromules in GFP-TUA6 (green) transgenic *N*. *benthamiana* leaf epidermal cells. Images are 6 representative images in indicated time points from electronic supplementary material, Movie S2. When *N. benthamiana* leaf epidermal cells are infected by *Pseudomonas syringae*, stromules are vigorously induced and attached to the nucleus. Dynamic stromule retractions bring about chloroplast body movement toward the nucleus (yellow arrows) and extension of stromules also occurs to withdraw the chloroplast body from the nucleus (white arrowheads), controlling the extent of the PNC during plant immunity.

Recently, it has been proposed that stromules might function to aid the dynamics of plastid–nuclear complexes leading to programmed cell death (PCD) in plant immunity [15]. Stromules are tubular protrusions stretched from the plastid body filled with stroma ([16,17]; figures 1*c* and 2). Recent studies unveiled a potential role of stromules as a path to transfer signalling molecules from plastids to the nucleus [13,17] and a regulatory factor to maintain the resulting plastid–nuclear complex via actin filaments during PCD [15]. Dynamic stromule formation is regulated differentially by actin and microtubule cytoskeletons [15,17]. Recently and interestingly, the causative defective gene in an *Arabidopsis* mutant displaying enhanced stromule formation in epidermal plastids was shown to be *PARC6* [18], which is also implicated in the formation of plastid–nuclear complexes in mesophyll cells (see above). Unlike the chloroplast body that primarily moves along actin filaments [3,10,11], stromules use microtubules as their guide to undergo directional extension and retraction. Interestingly, actin filaments provide anchor points to regulate stromule length [15], showing that movement of the chloroplast body and stromules are not regulated in the same manner. Interestingly, during immune responses, numerous stromules were observed to extend towards the nucleus and often wrap around the nucleus [13]. In addition, the tips of stromules can anchor to the periphery of the nucleus followed by a retraction of the stromules resulting in movement of the chloroplast body closer to the nucleus (figure 2; electronic supplementary material, Movie S2). This movement might be one of the mechanisms to maintain the plastid–nuclear complex observed in plant immunity [15]. However, genetic components to regulate stromules have yet to be identified. Although chloroplast body movement is altered in *chup1* [3,12,14], stromules were hyper-induced without pathogen infection in *N. benthamiana* cells showing suppressed *CHUP1* expression by RNA interference [13]. These data suggest that CHUP1 is a negative regulator of stromule formation. In these experiments, chloroplast bodies were frequently clustered similar to the plastid–nuclear complexes described above (figures 1*c* and 2), although unfortunately nuclei were not co-visualized [13]. Nevertheless, these data also suggest that CHUP1, presumably with as yet unidentified components, may also provide stromule-actin connectivity and stromule-mediated chloroplast movement towards the nucleus.

## 4. Problems of specificity

In almost all figures illustrating retrograde signalling, a single chloroplast is often depicted as the source of signals transduced to the nucleus (e.g. [1]). The reality in all higher plants' cells is somewhat different; multiple chloroplasts in cells are universal. Furthermore, in response to both internal and external cues it can be expected that not all chloroplasts inside a cell experience the same interaction with the environment. This is especially so for the light environment, where the light avoidance response (see above; [10]) results in stacking of chloroplasts and ensures that some experience higher light intensity than others. Therefore, from a signalling context, it is feasible that not all chloroplasts in a HL-exposed cell will communicate with the nucleus to the same degree. Thus, how could signalling from multiple chloroplasts be integrated by the nucleus to produce a defined change in gene expression? Likewise, for many small molecules or metabolites that also are signal transducers, more than one source in a cell is possible or likely. The exemplar is hydrogen peroxide (H_2_O_2_; [19]) with sources not only from the chloroplasts, but from the peroxisome, mitochondria, plasma membrane and cytosol [19,20]. In which case, how is it possible that an accumulation of H_2_O_2_ in nuclei but sourced from chloroplasts be distinguished, for example, from H_2_O_2_ sourced from peroxisomes? Finally, how would a metabolite acting as a signalling molecule avoid being diverted into another pathway *en route* to the nucleus from chloroplasts? The potential advantage of proximity or attachment of chloroplasts and nuclei is that any small molecule signal is directed to the nucleus so that chloroplast conditions are more specifically indicated. However, if metabolites first have to move to the cytosol they will very rapidly equilibrate across the cell. Therefore, for metabolites shared between chloroplasts and the cytosol, this could render them less effective as a chloroplast signal. Alternatively, compounds that are readily metabolized (e.g. H_2_O_2_) could be removed before entering the nucleus. This is illustrated by the ease of detecting photosynthesis-sourced H_2_O_2_ in nuclei but not cytosol in response to HL [4]. The starting point to answer to all of the above questions could be the spatial context in which signalling takes place in plastid–nuclear complexes. These complexes would allow direct communication between the origin of the transducing signal (the chloroplasts) and its destination (the nucleus). This is discussed further below, especially in the context of H_2_O_2_ as a transducing signalling molecule.

## 5. Partitioning and direct transfer of H_2_O_2_ from chloroplasts to the nucleus for signalling—a critical role for plastid–nuclear complexes and stromules?

In HL-exposed photosynthetically active cells, H_2_O_2_ accumulates in chloroplasts [4,21–26]. Various biochemical and genetic means of changing reactive oxygen species (ROS) levels in plant cells by promoting oxidative stress have been used to study the response of the transcriptome to H_2_O_2_ as well as other ROS (reviewed in [27]). The real value of the many independent transcriptomic datasets has been their combined study in meta-analyses using ever more statistically robust methodology [28,29]. This has provided strong evidence that a cohort of H_2_O_2_-responsive genes exist that are common to environmental and cellular cues, including exposure to HL. These meta-analyses do suggest that different subcellular sources of H_2_O_2_ could provide one element of signal specificity [27,29]. For example, a transcriptomics study of *Arabidopsis* genotypes with altered H_2_O_2_ production and scavenging capacities in chloroplasts and peroxisome respectively, which were shifted from non-photorespiratory to photorespiratory conditions, clearly indicated that the source of H_2_O_2_ may bring about a specificity of response [30]. In summary, specificity of H_2_O_2_ signalling is likely, but how would this be achieved? This is especially the case if we consider how H_2_O_2_ could be a retrograde signal transducer. The idea that H_2_O_2_ could convey a signal out of the chloroplast had been considered to be problematic [24,31]. The problem is that H_2_O_2_, in its supposed journey from the chloroplast to the nucleus, would not last long in the reducing environment of the cytosol. In addition, once it had exited the chloroplast, the source specificity of H_2_O_2_ would surely be lost. Consequently, the view was that H_2_O_2_ could initiate signalling but not onward transduction out of the chloroplast. Further signal transduction to the nucleus would have to be achieved by some other signalling molecule, which would be stable during its transit of the cytosol. However, subsequent research challenged this view. Isolated chloroplasts secrete H_2_O_2_ into their medium in a light intensity and photosynthetic electron transport (PET)-dependent manner [25] and there was the clear implication that this could also occur *in vivo*. Genetically encoded fluorescent protein biosensors that detect H_2_O_2_ enabled this question of its mobility and consequent specificity to be addressed [4,32]. These biosensors can detect H_2_O_2_ with a high degree of specificity in real-time, non-invasively and quantitatively [4,33–35]. Using such a probe (Hyper; [33]) expressed transiently in *N. benthamiana* abaxial epidermal cells and targeted to chloroplast stroma, cytosol and nucleus revealed that under HL, H_2_O_2_ levels increased in nuclei concomitant with the rates of accumulation in the chloroplast stroma [4]. The HL-dependent increase in H_2_O_2_ (measured as increased HyPer oxidation) in both compartments was dependent upon active PET. Furthermore, attenuation of the HL-triggered H_2_O_2_ accumulation in the chloroplast stroma by over-expressing the H_2_O_2_-scavenging enzyme ascorbate peroxidase (APX) also crucially inhibited its accumulation in the nucleus. This demonstrated that the H_2_O_2_ accumulation in the nucleus was directly dependent upon its accumulation in the chloroplast. The simplest, but not only, explanation for these observations is that transfer of H_2_O_2_ from chloroplasts to the nucleus occurs rapidly upon exposure to HL. Importantly, when a cytosolic isoform of APX was over-expressed it did not significantly attenuate accumulation of H_2_O_2_ in the nucleus. It was concluded that chloroplast-sourced H_2_O_2_ does not transit the cytosol and is a direct transfer from chloroplasts to the nucleus. In these abaxial epidermal cells, plastid–nuclear complexes are readily observed, consisting of a median 7 chloroplasts per nucleus, and it was noted that the oxidation of the HyPer probe in perinuclear clustering (PNC) chloroplasts was less than in those not associated with the nucleus. It was therefore hypothesized that it is the chloroplasts in plastid–nuclear complexes that transfer H_2_O_2_ directly to the nucleus. Interestingly, it has been previously observed [2] that chloroplasts detached from the nucleus underwent more rapid loss of chlorophyll fluorescence compared with those associated with the nucleus, implying different metabolic states for sub-populations of chloroplasts.

The same treatments that attenuated H_2_O_2_ in chloroplasts and nuclei also impacted on the expression of a *N. benthamiana* HL-responsive gene, *NbAPX1c*, in the same way, establishing that the H_2_O_2_ in the nucleus initiates onward signalling leading to the change in expression of at least one HL-responsive gene [4].

While the hypothesis of a direct transfer of H_2_O_2_ from chloroplasts to the nucleus is the simplest explanation of the data, other, not necessarily mutually exclusive, variations on this retrograde signalling mechanism remain possible. It is clear that chloroplast-sourced H_2_O_2_ initiates and drives the signalling and that HL-dependent accumulation of H_2_O_2_ in the nucleus continues that signalling process. Nor is the notion of a spatial dependence of signalling negated. However, it is conceivable that another signalling molecule is transferred to the nucleus that stimulates H_2_O_2_ synthesis in that compartment, or even that chloroplast-sourced H_2_O_2_ amplifies or activates its nuclear-localized synthesis. For example, nuclear-located cryptochromes (CRYs) when challenged with high fluence blue light can make H_2_O_2_ [36] and *CRY1* has been shown to positively regulate HL-responsive genes that are also responsive to H_2_O_2_ and require active PET [37,38].

H_2_O_2_ is also known to be generated and have a critical role as a signalling molecule to induce plant immunity [39]. When PCD occurs, chloroplasts function as a major generator of H_2_O_2_, which often induces gene expression in the nucleus [40]. Moreover, application of exogenous H_2_O_2_ to leaves has been shown to increase stromule formation [41]. Recently, H_2_O_2_ translocation from chloroplast to the nucleus *via* stromules has been raised as a possibility from work using the HyPer H_2_O_2_ sensor [13]. In live cell time-lapsed images, the concentration of H_2_O_2_ increased in stromules whose tips were anchored to the nucleus. In addition, by using nuclear-localized HyPer, an increase in H_2_O_2_ in the nucleus of plastid–nuclear complexes was monitored. Although these two events were monitored in separate experiments, this study does support the hypothesis that H_2_O_2_ is a retrograde signalling molecule in plant immunity. However, more sophisticated experiments will be required to monitor H_2_O_2_ translocation from chloroplasts into the nucleus in the same cell, in order to be able to propose that stromules might be a major path for H_2_O_2_-mediated retrograde signalling in plant immune responses.

Application of exogenous H_2_O_2_ is sufficient to induce stromule formation vigorously [13,41,42]. Furthermore, evidence has recently been presented that the establishment of pathogen- or effector-triggered immunity or treatment with H_2_O_2_ also causes the chloroplasts of *N. benthamiana* epidermal pavement cells to cluster around the nucleus [43]. Interestingly though, the authors did not report the presence of stromules during their observations. In summary, evidence may be emerging that H_2_O_2_ produced not only by chloroplasts but from other subcellular sources may also promote formation of both plastid–nuclear complexes and stromules. This implies a complex regulatory system, which we are just beginning to understand. However, all these observations have used agro-infected *N. benthamiana*, which might result in an interaction between HL and pathogen-associated molecular pattern (PAMP) responses [4,13,43] and therefore such observations do need to be confirmed in other experimental systems. Furthermore, some plant–pathogen interactions (e.g. that of *Arabidopsis* and *Pseudomonas syringae* DC3000) may suppress photosynthesis and chloroplast-sourced ROS in an effector dependent manner [44]. In this case, the impact of suppression of chloroplast function and ROS formation on stromule formation is unknown.

## 6. Retrograde signalling and H_2_O_2_ in cytosol microdomains

The close associations between chloroplasts and nuclei do not exclude retrograde signalling involving H_2_O_2_ also going *via* the cytosol and still achieving signalling specificity. Under HL, *N. benthamiana* abaxial epidermal cells do accumulate H_2_O_2_ in the cytosol but it is not evenly distributed. It must be assumed that the rate of diffusion of H_2_O_2_ from chloroplasts that are not part of plastid–nuclear complexes is sufficient to overcome rates of reducing activity from antioxidant systems in the cytosol for long enough to allow oxidation of the cytosol-located HyPer probe [4]. Active transport, i.e. secretion of H_2_O_2_ from chloroplasts, cannot be ruled out but no evidence is available on this point. If the resulting H_2_O_2_ microdomains are involved in signalling, then there would be temporal and spatial constraints meaning that redox-sensitive signal transducers will have to be in place to meet this localized H_2_O_2_ exiting from chloroplasts. There are candidate signal transducers that could act in such a role provided their spatial distribution in relation to H_2_O_2_ microdomains could be confirmed. At least three *Arabidopsis* heat shock transcription factors (HSFs), HSFA1D, HSFA8 and HSFA4A, have been shown to be redox-regulated [45–47]. Inter- and intramolecular disulfide bond formation is important in the conversion of inactive cytosol-located monomeric HSF isoforms into active trimeric forms that migrate to the nucleus to carry out their function. The high degree of sequence conservation in extensive plant HSF gene families suggests that such potential redox regulation may extend beyond these three examples [48] Signal transduction involving H_2_O_2_ in eukaryotes may involve the transfer of oxidising equivalents by thiol peroxidases (TPXs; [19,46]), which again would be required to be located or translocate to where H_2_O_2_ accumulates in microdomains. A simpler outcome could be that H_2_O_2_ from such chloroplasts, were it to continue to accumulate in the cytosol for any length of time, would lead to cellular oxidative stress and trigger PCD [31].

In summary, regarding the role of H_2_O_2_ as a signal transducer in retrograde signalling, there are clear layers of spatial dependency—plastid–nuclear complexes, stromules and microdomains. The juxtaposition of the players, once identified, in these signal transduction routes with respect to one another and to the accumulation of H_2_O_2_ will be critical in determining how H_2_O_2_-mediated retrograde signalling truly functions.

## 7. Spatial considerations of metabolites as retrograde signal transducers

As with H_2_O_2_, there are a myriad of small molecules that have single or distinct pools in chloroplasts and that are translocated to other parts of the cell as part of their normal role in cellular metabolism. Any molecule with a distinct origin or location in plastids has, therefore, the potential to be co-opted as a transducer in retrograde signalling. Recent productive lines of research have established at least 3 such metabolites or metabolic intermediates that fall into this class: 3**′**-phosphoadenosine 5**′**-phosphate (PAP; [49]) with cytosolic and chloroplast pools; methylerithrytol phosphate (MEcPP; [50]), a biosynthetic intermediate in plastid isoprenoid production; and β-cyclocitral, an oxidation product of carotenoids formed in chloroplasts [51]. These molecules have all been firmly established in the pantheon of prominent players in retrograde signalling. They have been proposed, and evidence offered, to be able to transduce signals out of the chloroplast and have been shown to strongly influence both whole-plant responses to environmental stress and the expression of a distinct cohort of genes [49–51]. To our knowledge, no spatial relationship between chloroplasts and the nucleus has been invoked as necessary for their signalling roles to be effective. However, clearly the workings of these signalling pathways could be enhanced if they were functioning in plastid–nuclear complexes or require stromules. For such spatial relationships to be established, specific genetically encoded biosensors would be needed to allow the necessary investigations to be done. The availability of such probes may still be some way off but would surely be of value to progress this field.

## 8. Spatial considerations of proteins as retrograde signal transducers

In contrast to the scores of metabolites and hormones that have been proposed as retrograde signal transducers, only a small number of proteins known to be targeted to the chloroplast have been identified subsequently in the nucleus to function as retrograde signal transducers in response to biotic and abiotic stresses. WHIRLY1 has been proposed to convey the redox status in chloroplasts to the nucleus in a salicylic acid-dependent manner [52]. WHIRLY1 proteins localize to both chloroplast and nucleus [53,54]. Expression of WHIRLY1 protein without its N-terminal plastid transit peptide sequence resulted in localization in the nucleus and successfully rescued the *whirly1* mutant phenotype [54]. Although dual localization of WHIRLY1 has been shown by several different approaches, how the translocation of WHIRLY1 from chloroplasts to the nucleus might occur is still not understood. An interesting chloroplast outer envelope protein, PTM (a PHD-type transcription factor with transmembrane domains) was proposed to translocate to the nucleus to regulate HL-responsive gene expression [55]. This translocation of PTM was proposed to allow its binding to the promoter of *ABSCISIC ACID INSENSITIVE4* to induce expression of light-responsive genes [55] and to the promoter of *FLOWERING LOCUS C* to control flowering under HL [56]. However, the identity of the signal from the chloroplast to induce an intramembrane proteolytic cleavage of the PTM is unknown and how the N-terminal moiety of the PTM is released from the chloroplast and finally ends up in the nucleus remains to be investigated. Subsequently, doubt about this proposed role of PTM was raised by the lack of impairment of a genomes-uncoupled phenotype in *ptm* mutants treated with norflurazon and lincomycin [57].

Several GFP-tagged proteins have shown to be present in stromules (e.g. carbonic anhydrase) and GFP photoconversion and photobleaching experiments suggest this is a dynamic process and that transfer of proteins between plastids can occur *via* stromules (reviewed in [17]). Recent studies of NUCLEAR RECEPTOR INTERACTING PROTEIN 1 (NRIP1) translocation from chloroplasts to nuclei *via* stromules might aid an understanding of the mechanism of translocation [13]. NRIP1 is a helper of N protein, which recognizes the p50 protein of TMV (tobacco mosaic virus) and, in turn, rapidly triggers plant immunity [58]. NRIP1 protein is localized in the stroma of chloroplasts of tobacco plant cells in normal conditions. However, upon TMV infection, NRIP1 proteins can translocate into the nucleus through stromules anchored to the nucleus [13].

Without further experimental support, it is hard to propose whether any of these above exemplar proteins translocate through stromules or directly by the plastid–nuclear complexes. However, given the proposed role of the stromules and the plastid–nuclear complexes to provide a path to transfer signalling molecules from chloroplasts to the nucleus in response to rapid changes of environmental status, it would be worth examining levels of stromules and the frequency of plastid–nuclear complexes in the WHIRLY1, PTM and NRIP1 activation conditions and their translocation *via* stromules and/or the plastid–nuclear complexes.

## 9. A time for stromules and a time for plastid–nuclear complexes: is the link photosynthesis?

Both plastid–nuclear complexes and stromules are now proposed to provide a spatial element to retrograde signalling. Especially in the case of H_2_O_2_-mediated retrograde signalling, such direct contacts between chloroplasts and their nucleus provide signalling specificity and permit this ROS to be a direct carrier of a signal from chloroplasts to their associated nucleus. Exactly the same argument and evidence are provided for stromules regarding H_2_O_2_-mediated retrograde signalling. The difference in signalling roles between plastid–nuclear complexes and stromules may be that the latter are able to provide a specific conduit for a much wider range of signalling molecules from the chloroplasts, including proteins [17]. However, to our knowledge, no evidence is available that protein-mediated retrograde signalling is definitively limited to stromules.

We have considered that plastid–nuclear complexes potentially provide a spatial component for signalling without stromules, but some studies show a high degree of stromule-producing chloroplasts present in such structures and stromules apparently facilitating the entry of their chloroplast into close contact with the nucleus (figure 2; electronic supplementary material, Movie S2). All of this points to a distinct function for stromules over and above any signalling role that is also achieved by direct contact between chloroplasts and the nucleus.

We have commented above that that some researchers observed stromules in their experimental systems and others have not. This suggests that specific physiological states of chloroplasts and cells are important in determining the circumstances that give rise to stromule formation. While the predominance of observations has been made in cells undergoing PCD, either as senescence or in the induction of pathogen- or elicitor-induced immunity (see above), it is premature to assume that stromule formation is a phenomenon linked to this process. This is because drought, salinity, phosphate limitation and ABSCISIC ACID (ABA) (possibly *via* strigolactone signalling) can also induce stromule formation [41,59] and these treatments, to our knowledge, do not induce PCD. Furthermore, isolated chloroplasts have been reported to be able to form stromules [60,61]. We propose instead that all these situations have in common a diminution in photosynthesis and primary metabolism. Induction of drought stress or phosphate limitation, and more controversially, exogenous ABA often disrupt photosynthesis [62–64]. The impact of immunity and senescence on photosynthesis is always associated with a decline in this function [44,65]. Furthermore, any restriction of photosynthesis and consequent rise in the oxidation state of the stroma is a likely pre-requisite for stromule formation [60].

## Supplementary Material

Changing views of PNCs in N. benthamiana abaxial epidermal cells.

## Supplementary Material

Stromules mediate chloroplast movement towards and away from the nucleus

## Data accessibility

This article has no additional data.
